# Atrial Fibrillation is A Predictor of Drug-Related Bradycardia and Hospital Admission in Older Adults

**DOI:** 10.14336/AD.2021.0208

**Published:** 2021-08-01

**Authors:** Charlotte Griffiths, Adam Ioannou, Benjamin Dickinson, Sofia Metaxa, Fouad R Amin, Amit K J Mandal, Constantinos G Missouris

**Affiliations:** ^1^Wexham Park Hospital, Frimley Health NHS Foundation Trust, UK; ^2^Royal Free Hospital, Royal Free London NHS Foundation Trust, UK; ^3^University of Cyprus Medical School, Nicosia, Cyprus

To the editor:

Drug-related bradycardia (DRB) is a common conundrum in clinical practice and is often an adverse effect of a desired effect. However, it can result in multiple hospital admissions, particularly for older adults.

Over a three-month period, we identified 100 hospitalised patients aged over 70 years with 2 or more documented episodes of bradycardia during admission (defined as heart rate of less than 60 beats per minute during the daytime - nocturnal bradycardic episodes were not deemed significant). These constituted 4.32% of total admissions. Syncope was the most common presenting symptom and occurred in 23 patients. Causality assessment via Naranjo algorithm demonstrated a definite causal association between the administration of rate-limiting medications and bradycardia (score of 9) [1]. Beta-blockers were the most commonly prescribed agent (54 patients) and sinus bradycardia was the most commonly identified rhythm disturbance (41 patients) on resting 12 lead electrocardiogram (ECG). 26 patients had paroxysmal or permanent atrial fibrillation (AF) (of which 9 had documented heart failure [heart failure reduced ejection fraction = 4; heart failure preserved ejection fraction = 5; 1 patient had both], 14 had underlying coronary artery disease, 4 had hypertension, 2 had type 2 diabetes mellitus, and 3 had chronic obstructive pulmonary disease). AF was identified as a significant predictor of DRB (OR=10.2, 95% CI 3.3 - 31.6, P<0.001). There were no other significant differences between the baseline gender and comorbidities of patients who had DRB and those who did not.

## 17 (65.4%) of the patients with AF had an up-to-date transthoracic echocardiogram. We assessed left atrial diameter as a surrogate marker for chronicity and burden of AF and found severely enlarged left atria in all but 1 patient; however, there was no statistically significant difference between those who had DRB and those who did not (63 mm ± 13mm vs. 61mm ± 7mm, P=0.712).

Although our study did not report any in-hospital death, during the 12-month follow-up period 30 patients had deceased, 14 patients were re-admitted with DRB and 5 patients underwent permanent pacemaker implantation. Neither an admission with DRB or a background of AF were significant predictors of mortality.

Patients initially diagnosed with AF often have a rapid ventricular response and require rate limiting medications such as beta-blockers and non-dihydropyridine calcium channel blockers drugs to mediate their heart rate As AF progresses there is electrical remodelling and changes in the expression of ion channels within the cardiomyocytes leading to myocardial fibrosis This in turn slows conduction through cardiomyocytes and leads to delayed ventricular response or “slow AF” This process occurs over many years and can remain clinically silent [2,3] We suspect DRB may “unmask” chronotropic incompetence from underlying sinoatrial and/or atrioventricular node dysfunction, and this is supported by many studies demonstrating that therapeutic doses of these agents do not cause symptomatic bradycardia in structurally normal hearts Notably, older patients are more likely to have other concomitant cardiovascular comorbidities that can impact on cardiac architecture and performance, and these often require treatment with negatively chronotropic agents.

Without pharmacovigilance older adults may continue taking rate-limiting drugs despite a gradual reduction in their heart rate. Another point to underscore, is that many older adults are underweight and sarcopenic (especially the poly-morbid) which can result in prescription errors based on weight and size. Caution should therefore be exercised when initiating and up-titrating rate-limiting medications in older patients, and those with AF should undergo regular review of their heart rate and ECG surveillance. Holter monitoring may be a valuable tool within in this context and should be routinely employed.
